# Analysis of metabolic effects of menthol on WFS1‐deficient mice

**DOI:** 10.14814/phy2.12660

**Published:** 2016-01-05

**Authors:** Marite Ehrlich, Marilin Ivask, Atso Raasmaja, Sulev Kõks

**Affiliations:** ^1^ Department of PathophysiologyCentre of Translational MedicineUniversity of TartuTartuEstonia; ^2^ Department of PhysiologyCentre of Translational MedicineUniversity of TartuTartuEstonia; ^3^ Division of Pharmacology and PharmacotherapyFaculty of PharmacyUniversity of HelsinkiHelsinkiFinland

**Keywords:** Menthol, metabolism, *Trpm8*, Wfs1

## Abstract

In this study, we investigated the physiological regulation of energy metabolism in wild‐type (WT) and WFS1‐deficient (Wfs1KO) mice by measuring the effects of menthol treatment on the O_2_ consumption, CO_2_ production, rectal body temperature, and heat production. The basal metabolism and behavior was different between these genotypes as well as TRP family gene expressions. Wfs1KO mice had a shorter life span and weighed less than WT mice. The food and water intake of Wfs1KO mice was lower as well as the body temperature when compared to their WT littermates. Furthermore, Wfs1KO mice had higher basal O_2_ consumption, and CO_2_ and heat production than WT mice. In addition, Wfs1KO mice showed a higher response to menthol administration in comparison to WT mice. The strongest menthol effect was seen on different physiological measures 12 h after oral administration. The highest metabolic response of Wfs1KO mice was seen at the menthol dose of 10 mg/kg. Menthol increased O_2_ consumption, and CO_2_ and heat production in Wfs1KO mice when compared to their WT littermates. In addition, the expression of *Trpm8* gene was increased. In conclusion, our results show that the Wfs1KO mice develop a metabolic phenotype characterized with several physiological dysfunctions.

## Introduction

The first description of Wolfram syndrome (WS), an autosomal recessive disorder, was reported in 1938. The acronym DIDMOAD summarizes the most frequent findings: diabetes insipidus, diabetes mellitus, optic atrophy, and deafness. The minimal criteria for diagnosis are diabetes mellitus and optic atrophy (Strom et al. 1998).

The prevalence of children and adolescents aged under 21 years with WS in Europe is 33/17,000,000 (~1:500,000), and the prevalence of children and adolescents aged under 21 years with WS and diabetes is 33/24,000 (~1:730) (Rohayem et al. 2011). The cause of WS is the loss‐of‐function mutations in wolframin (*Wfs1*) gene (Strom et al. 1998). *Wfs1* is linked to the short arm of chromosome 4 p16.1 (Polymeropoulos et al. 1994; Collier et al. 1996). The *Wfs1* gene encodes wolfamin (WFS1), a protein with 890 amino acid residues and a molecular mass of 100 kDa. WFS1 is a hydrophobic glycoprotein which contains nine transmembrane segments, with the N‐terminus localized in the cytoplasma and the C‐terminus in the endoplasmic reticulum lumen (Hofmann et al. 2003).

WFS1 protein is expressed in the brain, heart, lungs, and pancreatic *β*‐cells (Inoue et al. 1998; Strom et al. 1998; Hofmann et al. 2003; Ishihara et al. 2004). In the brain, WFS1 is expressed in regions related to the neurological disabilities. Prominent expression was evident in the hippocampus, amygdala, olfactory tubercle, allocortex, ventral striatum, prefrontal cortex, and proisocortical areas (Takeda et al. 2001; Luuk et al. 2008). WFS1 expression sites suggest that WFS1 may function in emotional, behavioral, and visceral control (Takeda et al. 2001).

Transient receptor potential melastatin 8 (TRPM8) and transient receptor potential vanilloid 3 (TRPV3) are members of the TRP ion channel family (McKemy et al. 2002). TRPM8 channels are activated by cold (8–28°C) and chemicals, for example menthol, which induce cold sensation, positive membrane potential, and activities of endogenous signaling lipids like phosphatidylinositol‐4,5‐bisphosphate (PIP_2_) (McKemy et al. 2002; Peier et al. 2002; Nilius et al. 2007). TRPV3 is being activated by temperature >32–39°C or by chemical stimuli, for example menthol (Xu et al. 2002; Macpherson et al. 2006). McKemy et al. (2002) demonstrated that TRP ion channels are used as primary transducers of thermal stimuli for thermosensation mediated by common molecular mechanism. Analyzing our RNA sequencing results, we found increased expression of *Trpm8* and *Trpv3* in the hippocampus of Wfs1KO mice and therefore hypothesized changed sensitivity in Wfs1KO mice to the metabolic effects of menthol (Table S1).

The aim of this study was to compare the metabolic differences and dose–response effects to menthol between WFS1‐deficient mice and their wild‐type (WT) littermates.

## Methods

### Animals

WFS1‐deficient (Wfs1KO) mice were generated by invalidating the eighth exon of the *Wfs1* gene (Luuk et al. 2008). Experiments were performed with 9–12 months old male F2 hybrids (129S6/SvEvTac x 129S6/SvEvTac). Mice were kept in groups of eight per cage at 22 ± 1°C in a room illuminated artificially from 7 am to 7 pm. Tap water and food pellets were freely available. The permission (No. 71, 8 April 2011) for this study was given by the Estonian National Board of Animal Experiments in accordance with the European Communities Directive of 24th of November, 1986 (86/609/EEC). Metabolic experiment was carried out with naive animals. Wfs1KO mice were always used in parallel with their WT littermates and the animals were randomly divided into experimental groups.

### Metabolic studies

Control group animals in the metabolic analysis received an oral administration of water. l‐menthol (W266523; Sigma‐Aldrich, St. Louis, MO, USA) was dissolved in water on a heating plate at 40°C. l‐menthol was stirred before oral gavage administration of doses of 8, 10, 15, and 20 mg/kg. Different doses were administrated in a decreasing order on four consecutive days. l‐menthol was orally administered at a volume of 200 *μ*L/30 g.

Eight Wfs1KO mice and eight WT mice were used in the study with 8 mg/kg l‐menthol. Twelve Wfs1KO and twelve WT mice were used in the menthol study with 10, 15, and 20 mg/kg doses. There were five Wfs1KO mice and eight WT mice in the control group. Mice were kept 1 week before metabolic measurement alone in cages. After adaption period, the mice were studied in the metabolic cages (TSE Phenomaster; TSE Systems GmbH, Bad Homburg, Germany). Basal data were measured during the first 2 days in metabolic cages. The basal data of second day were used in the analysis. Within the next 4 days, once per day one of the following menthol dose 8, 10, 15, or 20 mg/kg was given. Metabolic effect was measured 24 h after oral menthol administration. For the next 4 days after 2 days of basal data measurements, 0.2 mL of water was administrated by oral gavage to the control group once per day. Rectal temperature (°C) was measured 1 and 2 h after oral menthol administration. Food (g), water (mL), average O_2_ consumption (mL/h/kg), average CO_2_ production (mL/h/kg), and average heat production (kcal/h/kg) data were collected over time period of 24 h for each dose. Weight change data were collected over time period of 4 days. The metabolic data of mice were analyzed 3, 7, 12, and 14 h after oral administration of menthol. Metabolic cages automatically measured and software calculated energy consumption H (kcal/kg/h), taking into account 100% the weight of the mouse. The average respiratory coefficient was calculated based on the average CO_2_ eliminated divided with the average O_2_ consumed at certain time point. Weight change was measured after 4 days of menthol administration to oral gavage.

### Statistics

The results of the metabolic analysis are expressed as mean values ± SEM. Weight change, food consumption, and drink consumption are expressed as mean values ± SD. Mann–Whitney *U*‐test was applied for the statistical analysis of metabolic data. For survival analysis, we used Kaplan–Meyer estimator. *P*‐value lower than 0.05 (*P* < 0.05) was considered statistically significant. Statistical analysis was done with statistical computing program R software (http://www.r-project.org/).

## Results

### Life span

**Figure 1:**
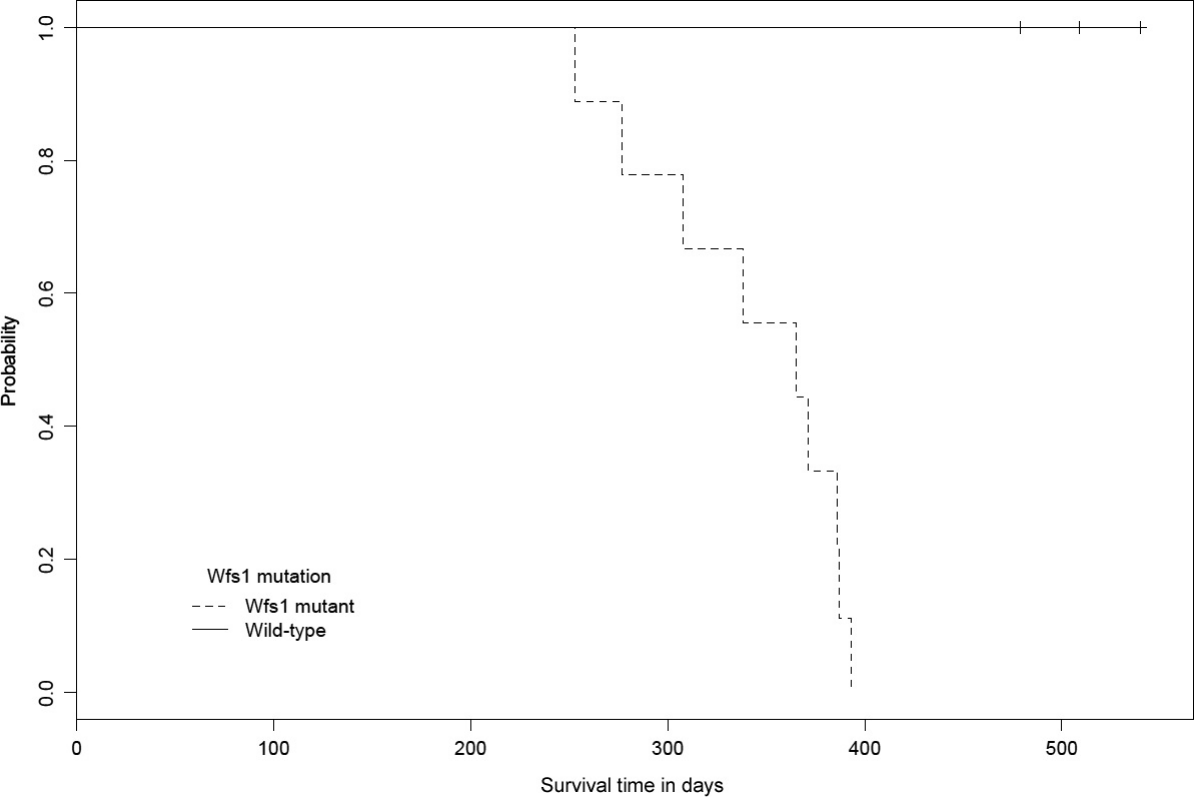
Comparison of life span between mice of different genotypes. Wfs1KO mice have a shorter life span compared to WT mice. N_W__fs1_ _KO_ = 9, N_WT_ = 8.

Wfs1KO mice have a shorter life span compared to WT mice (*P* < 0.05) (Fig. 1). Average life span of Wfs1KO was 11 months and that of WT mice 15–16 months.

### Body weight

The general physiological data are presented in the Table 1. The body weights were statistically different between genotypes. It was found that Wfs1KO mice (Weight_Wfs1KO_ = 18.38 ± 2.50 g) weigh less compared to WT mice (Weight_WT_ = 27.85 ± 3.47 g) (Table 1). Wfs1KO mice did not lose statistically more body weight after menthol treatment compared to WT mice (Table 1). Wfs1KO mice moderately remained or gained body weight after menthol treatment (Weight_Wfs1KO_ = 0.22 ± 2.44 g) compared to WT mice, which moderately lost body weight after menthol treatment (Weight_WT_ = −0.25 ± 1.33 g).

**Table 1:** Average data of basal and menthol treatment of different metabolic parameters

	Wfs1KO	WT
Weight (g)	18.38 ± 2.50	27.85 ± 3.47^c^
Weight change (Δg)	0.22 ± 2.44	−0.25 ± 1.33
Food consumption (g)	1.48 ± 1.03	2.54 ± 0.94^c^
Food consumption (Δg)	0.2 ± 0.34	0.24 ± 0.58
Water consumption (mL)	1.76 ± 2.21	2.59 ± 1.58^a^
Water consumption (ΔmL)	−0.38 ± 0.23	0.36 ± 0.18
Body temperature (°C)	35.76 ± 2.24	36.86 ± 1.25^a^
Body temperature change after 1 h menthol administration (Δ°C)	0.57 ± 2.24	0.65 ± 1.25
Body temperature change after 2 h menthol administration (Δ°C)	0.68 ± 2.24	0.77 ± 1.25
O_2_ consumption (mL/h/kg)	7290 ± 1816^c^	5435 ± 1108
CO_2_ production (mL/h/kg)	6827 ± 2177^b^	5516 ± 1352
Heat production (kcal/h/kg)	36.07 ± 9.79^c^	27.47 ± 5.78
Respiratory coefficient 3 h after menthol administration	0.89	0.89
Respiratory coefficient 7 h after menthol administration	0.92	0.87
Respiratory coefficient 12 h after menthol administration	0.96	0.92
Respiratory coefficient 14 h after menthol administration	0.97	0.94

Data are expressed as mean values ± SD.

a *P* < 0.05.

b *P* < 0.005.

c *P* < 0.001.

### Food and water consumption

The food and water intake in Wfs1KO mice (Food_Wfs1KO_ = 1.48 ± 1.03 g, Water_Wfs1KO_ = 1.76 ± 2.21 mL) was significantly lower compared to WT mice (Food_WT_ = 2.54 ± 0.94 g, Water_WT_ = 2.59 ± 1.58 mL) (Table 1). After menthol treatment, Wfs1KO mice did not consume less food or water compared to WT mice (Fig. 1).

### Body temperature

The body temperatures were significantly higher in WT mice (36.86 ± 1.25°C) compared to Wfs1KO mice (35.76 ± 2.24°C) (Table 1). Wfs1KO and WT mice body temperature rose 1 and 2 h after menthol treatment, but the effect was not statistically significant. WT mice had statistically higher body temperature 1 and 2 h after menthol treatment compared to Wfs1KO mice. Interestingly, the effect of menthol on rising body temperature was absent 1 and 2 h after menthol treatment (Fig. S1).

### Oxygen consumption and carbon dioxide production

**Figure 2:**
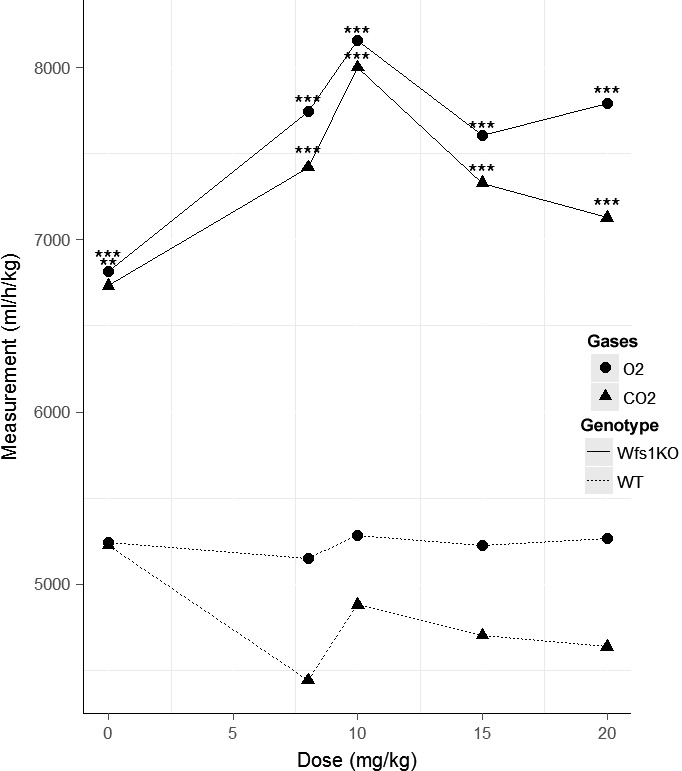
Oxygen consumption and carbon dioxide production comparison in Wfs1KO and WT mice 12 h after oral menthol administration. Wfs1KO mice oxygen consumption and carbon dioxide production is statistically higher after oral menthol administration of doses 8, 10, 15, and 20 mg/kg compared to WT mice. Statistically, highest effect on oxygen consumption and carbon dioxide production had menthol dose 10 mg/kg. Wfs1KO – Wfs1‐deficient mice, WT – wild‐type mice, O_2_ – oxygen consumption (mL/h/kg), CO_2_ – carbon dioxide production (mL/h/kg). *N* = 8–12 for each group. *P < 0.05, **P < 0.005, ***P < 0.001.

Wfs1KO mice (ΔO_2_ = 7290 ± 1816 mL/h/kg) had significantly higher basal O_2_ consumption and basal CO_2_ production (ΔCO_2_ = 6827 ± 2177 mL/h/kg) compared to WT mice (ΔO_2_ = 5435 ± 1108 mL/h/kg, ΔCO_2_ = 5516 ± 1352 mL/h/kg) (Table 1). The analysis of average O_2_ consumption and CO_2_ production after oral menthol administration showed that the effect on Wfs1KO mice was significantly highest 12 h after administration compared to WT mice. The strongest effect on Wfs1KO mice was with menthol dose 10 mg/kg (Fig. 2). Average O_2_ consumption rose significantly 3 and 7 h after menthol treatment. Average CO_2_ production rose significantly 3 and 12 h after menthol treatment. Menthol treatment increased the average O_2_ consumption (ΔO_2_ = 7875 ± 1816 mL/h/kg) and CO_2_ production (ΔCO_2_ = 7616 ± 2177 mL/h/kg) of Wfs1KO mice compared to WT mice, whose O_2_ consumption (ΔO_2_ = 5329 ± 1108 mL/h/kg) and CO_2_ production (ΔCO_2_ = 4988 ± 1352 mL/h/kg) decreased.

### Thermogenesis

**Figure 3:**
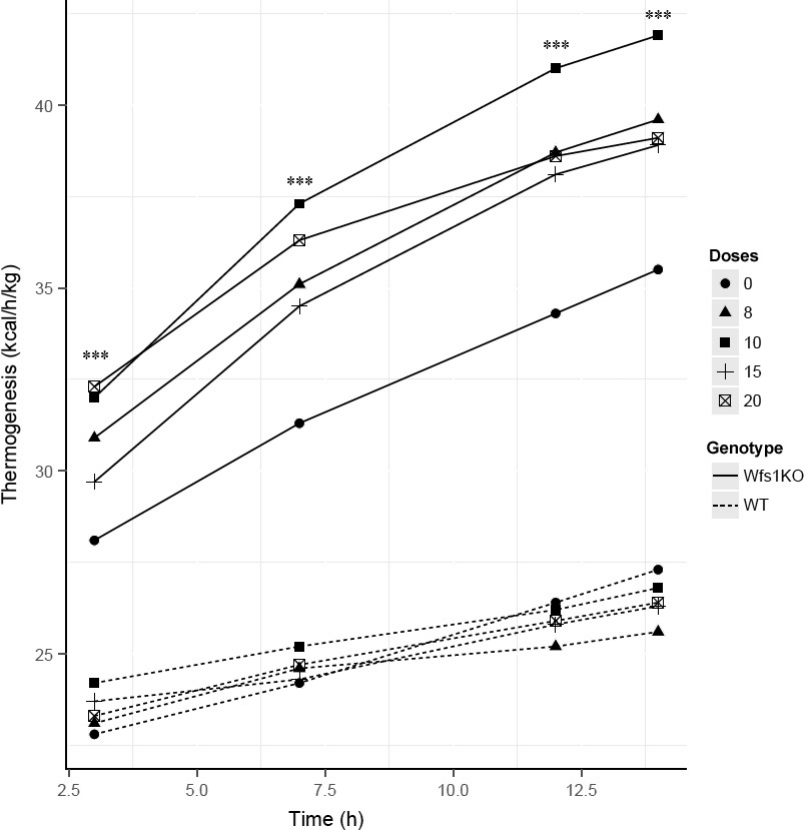
Menthol activated heat production in Wfs1KO and WT mice. Wfs1KO heat production is statistically higher after oral menthol administration of doses 8, 10, 15, and 20 mg/kg compared to WT mice. Statistically, highest effect on heat production had menthol dose 10 mg/kg. Wfs1KO – Wfs1‐deficient mice, WT – wild‐type mice, circle – mice heat production (kcal/h/kg) 3 h after oral menthol administration, triangle – mice heat production (kcal/h/kg) 7 h after oral menthol administration, square – mice heat production (kcal/h/kg) 12 h after oral menthol administration, cross – mice heat production (kcal/h/kg) 14 h after oral menthol administration. *N* = 8–12 for each group. *P < 0.05, **P < 0.005, ***P < 0.001.

Wfs1KO mice (H = 36.07 ± 9.79 kcal/h/kg) had significantly higher heat production compared to WT mice (H = 27.47 ± 5.78 kcal/h/kg) (Table 1). The average heat production after oral menthol administration showed that the effect on Wfs1KO mice was significantly highest 12 h after administration compared to WT mice. The strongest effect on Wfs1KO mice was with menthol dose 10 mg/kg (Fig. 3). Average heat production rose significantly 3 and 7 h after menthol treatment. Fourteen hours after menthol treatment, the average heat production of Wfs1KO mice was increased (H = 39.46 ± 9.79 kcal/h/kg) whereas that of WT mice was decreased (H = 26.52 ± 5.78 kcal/h/kg).

## Discussion

RNA sequencing showed that *Trpm8* and *Trpv3* genes were overexpressed in the hippocampus of Wfs1KO mice compared to WT littermates (Table S1). Warming the skin of TRPM8‐deficient (Trpm8KO) mice induces TRPM8 output, which indicates that the thermostat of the skin temperature against cooling acts TRPM8 (Tajino et al. 2011). The core temperature of Trpm8KO mice was lower in a room cooled from 27°C to 10°C compared to WT mice with a slightly increased core temperature. The skin temperature decreased by the same room cooling in both mice indicating that TRPM8‐induced thermogenesis in the core is not powerful enough to warm the skin against cooling (Tajino et al. 2011). Trpm8KO mice have only about 5% of all neurons that are cold sensitive and Trpm8KO mice cannot sense differences between warm (30°C) or cold (15°C) temperatures compared to WT mice (Bautista et al. 2007).

Noormets et al. (2014) showed that Wfs1KO mice weigh less and lose more weight in the end of metabolic experiment compared to WT mice. Koks et al. (2009) have showed the reduced body weight phenotype in Wfs1KO mice. Wfs1KO mice were much smaller and they had damaged glucose tolerance (Luuk et al. 2009). In this study, the body weight of Wfs1KO mice were significantly lower compared to WT mice. Ma et al. (2012) showed that the body weight was not different between mice on chow diet with and without menthol, although mice on dietary menthol had elevated activity and body temperature. Ma et al. (2012) confirmed that menthol mainly affected body fat, but not other compositions of body weight. In this study, there was no statistical significance to prove that Wfs1KO mice lost more body weight at the end of the experiment compared to WT mice after being treated orally with different doses of menthol.

It is important to investigate the changes in metabolism caused by WS, since patients with WS have neurodegenerative characteristics (Strom et al. 1998). Studies have shown that patients with Alzheimer's, Parkinsons's, or Huntington's diseases have impaired glucose metabolism, increased insulin resistance and abnormal appetite regulation (Cai et al. 2012). Patients with Huntington's disease show severe weight loss in spite of healthy appetite and high caloric intake (Morales et al. 1989; Trejo et al. 2004; Cai et al. 2012). Patients with Huntington's disease also have higher energy expenditure compared to controls (Pratley et al. 2000; Stoy and McKay 2000; Gaba et al. 2005; Cai et al. 2012). Patients with Parkinson's disease have the same characteristics (Cai et al. 2012). Cai et al. (2012) has suggested that there is a link between the progression of Huntington's disease and Parkinson's disease and metabolic dysfunction. This study shows that Wfs1KO mice have a shorter life span compared to WT mice. Restoring the metabolic homeostasis may improve cognitive and motor function, and also increase life span in patients with Alzheimer's or Huntington's disease (Watson et al. 2005; Martin et al. 2009).

Ma et al. (2012) showed that long‐term dietary menthol treatment had no effect on food intake in WT mice. The results in this study showed that Wfs1KO mice consume less food and water compared to WT mice. After menthol treatment, Wfs1KO mice did not consume less food or water compared to WT mice, which mean that the statistical significance between genotypes after menthol treatment disappeared.

Ma et al. (2012) showed that dietary menthol significantly increased oxygen consumption in WT mice, but not in Trpm8KO mice, suggesting that dietary menthol increased the resting metabolic rate through TRPM8 activation. Ma et al. (2012) results are in correspondence with the results of this study since Wfs1KO mice have overexpressed *Trpm8*, which might be the cause of higher basal O_2_ consumption and CO_2_ production compared to WT mice.

Kozyreva et al. (2013) results showed that 1% suspension of menthol in saline increased O_2_ consumption and CO_2_ release less than 30% in response to warming. The respiratory coefficient did not change during warming. Under thermoneutral conditions, menthol activation of TRPM8 reduced the respiratory coefficient by enhancing O_2_ consumption by 10% at constant CO_2_ release (Kozyreva et al. 2013). In this study, menthol treatment increased the average respiratory coefficient of Wfs1KO mice and decreased the average respiratory coefficient of WT mice and recovered 12 h after menthol treatment followed in increasing. This study also showed that the difference in O_2_ consumption and CO_2_ production between genotypes was biggest at 12 h after menthol administration and with menthol dose of 10 mg/kg. Kozyreva et al. (2013) results showed that O_2_ consumption and CO_2_ production did not differ after TRPM8 activation with menthol. In this study, the strongest menthol effect was seen with the oral dose of 10 mg/kg resulting in increased O_2_ consumption and CO_2_ production of Wfs1KO mice. Furthermore, the overall menthol treatment increased the average O_2_ consumption and CO_2_ production in Wfs1KO mice compared to WT mice showing an opposite response with the decreased average O_2_ consumption and CO_2_ production.

In this study, Wfs1KO mice had significantly higher basal heat production compared to WT mice. Noormets et al. (2014) studies showed that the mean heat production was not different between Wfs1KO and WT mice. Mean oxygen consumption was higher in Wfs1KO female mice compared to Wfs1KO male mice. No such difference was seen compared to the WT mice littermates neither between the sexes within the WT mice group (Noormets et al. 2014). The differences between two studies might be the age of the mice. Noormets et al. (2014) used 2–3 months old mice, but in this study 9–12 months old mice were used. The change in heat production of Wfs1KO mice was noticeable. In this study, menthol dose of 10 mg/kg raised significantly Wfs1KO mice heat production from the beginning of 3 h after oral menthol administration. The strongest effect on Wfs1KO heat production was at 10 mg/kg menthol dose 12 h after oral administration. Different menthol doses had low effect on WT mice or even lowered their heat production compared to control group.

Masamoto et al. (2009) measurements showed that administration of 20 mg/kg menthol increased both colonic temperatures, by inserting the thermistor 2 cm into the colon, and intrascapular brown adipose tissue temperature, by the thermistor placed between an intrascapular brown adipose tissue pad and the trapezius muscle. The tail‐skin temperature did not change after the infusion with TRPM8 agonists menthol or 1,8‐cineole. In this study, the average body temperature of Wfs1KO and WT mice increased 1 and 2 h after menthol treatment, but the results were not statistically significant between genotypes. The lack of menthol effect on body temperature change may be explained with Masamoto et al. (2009) results, which suggest that menthol‐induced thermogenesis did not have effect on heat diffusion. Ma et al. (2012) showed that dietary menthol markedly increased the locomotor activity and rectal temperature of WT mice, but the effect was almost absent in Trpm8KO mice. Measuring of 24 h ambulatory core body temperature, they found that during the day time, core temperatures were not different, but during the nocturnal, core temperatures were significantly higher in WT mice on the menthol diet compared with WT mice on the chow diet compared to Trpm8KO mice, whose core body temperature was not significantly higher either during nocturnal and day time. In this study, only the rectal body temperature was measured and the body temperature changes had no statistical significance 1 and 2 h after oral administration with different menthol doses, but WT mice have significantly higher body temperature 1 and 2 h after menthol treatment compared to Wfs1KO mice. Different menthol doses were orally administrated during day time and it is possible that 2 h after menthol administration the effect of menthol on body temperature was not apparent yet.

## Conclusions

Wfs1KO mice weigh less than WT mice indicating some metabolic disturbances in the mutant mice. Wfs1KO mice have also significantly higher O_2_ consumption, and CO_2_ and heat production compared to WT mice. The experiments revealed that the dose of 10 mg/kg of a TRPM8 agonist, menthol, is most effective by increasing the heat production, O_2_ consumption, and CO_2_ production of Wfs1KO mice compared to WT mice. Interestingly, menthol increased the metabolic parameters of Wfs1KO mice compared to WT mice, whose average metabolic parameters decreased after menthol treatment. The shorter life span, metabolic changes and overexpression of *Trpm8* gene in Wfs1KO mice suggest that Wfs1KO mice have serious metabolic dysfunctions. Further studies are in progress to clarify the mechanism of menthol and the role of *Trpm8* gene in the metabolism of Wfs1KO and WT mice.

## Conflict of Interests

The authors declare that the research was conducted in the absence of any commercial or financial relationships that could be construed as a potential conflict of interest.

## Supporting information




**Table S1.** Expression of TRP family genes in the hippocampus of Wfs1KO mice. RNA sequencing results for TRP family genes in the hippocampus of Wfs1KO mice compared to WT mice showed upregulation of *Trpm8* and *Trpv3* genes as log ratio of fold change (logC) >2 and false discovery rate (FDR) <0.05 was considered significant.

**Figure S1.** Effect of menthol treatment on body temperature in Wfs1KO and WT mice. Different menthol doses did not change the body temperature of Wfs1KO mice and WT mice after 1 and 2 h of oral administration. Wfs1KO – body temperature (°C) of Wfs1‐deficient mice, WT – body temperature (°C) of wild‐type mice. Circle – mice rectal body temperature 1 h after menthol treatment, triangle – mice mice rectal body temperature 2 h after menthol treatment. *P < 0.05, **P < 0.005, ***P < 0.001.
