# Supplementary material for: Analysis of metabolic effects of menthol on WFS1‐deficient mice
Source: Physiol Rep. 2016 Jan 5;4(1):e12660. doi: 10.14814/phy2.12660 (PMC4760410; doi:10.14814/phy2.12660)
Supplement: Supplementary file 1 — Table S1. Expression of TRP family genes in the hippocampus of Wfs1KO mice. RNA sequencing results for TRP family genes in the hippocampus of Wfs1KO mice compared to WT mice showed upregulation of Trpm8 and Trpv3 genes as log ratio of fold change (logC) >2 and false discovery rate (FDR) <0.05 was considered significant. [file PHY2-4-e12660-s001.docx]

**Supplementary data**

Table 2. Expression of TRP family genes in the hippocampus of Wfs1KO mice. RNA-sequencing results for TRP family genes in the hippocampus of Wfs1KO mice compared to WT mice showed upregulation of *Trpm8* and *Trpv3* genes as log ratio of fold change (logC) > 2 and false discovery rate (FDR) < 0.05 was considered significant.

| **Symbol** | **logFC** | **FDR** | **Entrez Gene Name** |
| --- | --- | --- | --- |
| *Trpm1* | 0.212 | 0.797 | transient receptor potential cation channel, subfamily M, member 1 |
| *Trpm2* | -0.455 | 0.033 | transient receptor potential cation channel, subfamily M, member 2 |
| *Trpm3* | 0.284 | 0.701 | transient receptor potential cation channel, subfamily M, member 3 |
| *Trpm4* | -0.212 | 0.632 | transient receptor potential cation channel, subfamily M, member 4 |
| *Trpm5* | 0.870 | 0.028 | transient receptor potential cation channel, subfamily M, member 5 |
| *Trpm6* | 0.358 | 0.491 | transient receptor potential cation channel, subfamily M, member 6 |
| *Trpm7* | 0.010 | 1 | transient receptor potential cation channel, subfamily M, member 7 |
| *Trpm8* | 6.624 | 1.44E-198 | transient receptor potential cation channel, subfamily M, member 8 |
| *Trpa1* | -0.522 | 0.410 | transient receptor potential cation channel, subfamily A, member 1 |
| *Trpc1* | -0.042 | 0.979 | transient receptor potential cation channel, subfamily C, member 1 |
| *Trpc2* | 0.170 | 0.930 | transient receptor potential cation channel, subfamily C, member 2 |
| *Trpc3* | -0.021 | 1 | transient receptor potential cation channel, subfamily C, member 3 |
| *Trpc4* | -0.244 | 0.370 | transient receptor potential cation channel, subfamily C, member 4 |
| *Trpc5* | -0.140 | 0.756 | transient receptor potential cation channel, subfamily C, member 5 |
| *Trpc6* | 0.083 | 0.869 | transient receptor potential cation channel, subfamily C, member 6 |
| *Trpc7* | 0.197 | 0.766 | transient receptor potential cation channel, subfamily C, member 7 |
| *Trpv1* | -0.070 | 1 | transient receptor potential cation channel, subfamily V, member 1 |
| *Trpv2* | -0.010 | 1 | transient receptor potential cation channel, subfamily V, member 2 |
| *Trpv3* | 2.255 | 8.15E-05 | transient receptor potential cation channel, subfamily V, member 3 |
| *Trpv4* | 0.679 | 0.526 | transient receptor potential cation channel, subfamily V, member 4 |
| *Trpv5* | -1.318 | 0.005 | transient receptor potential cation channel, subfamily V, member 5 |
| *Trpv6* | 0.710 | 0.226 | transient receptor potential cation channel, subfamily V, member 6 |
