# Supplementary figures and images for: Analysis of metabolic effects of menthol on WFS1‐deficient mice
Source: Physiol Rep. 2016 Jan 5;4(1):e12660. doi: 10.14814/phy2.12660 (PMC4760410; doi:10.14814/phy2.12660)

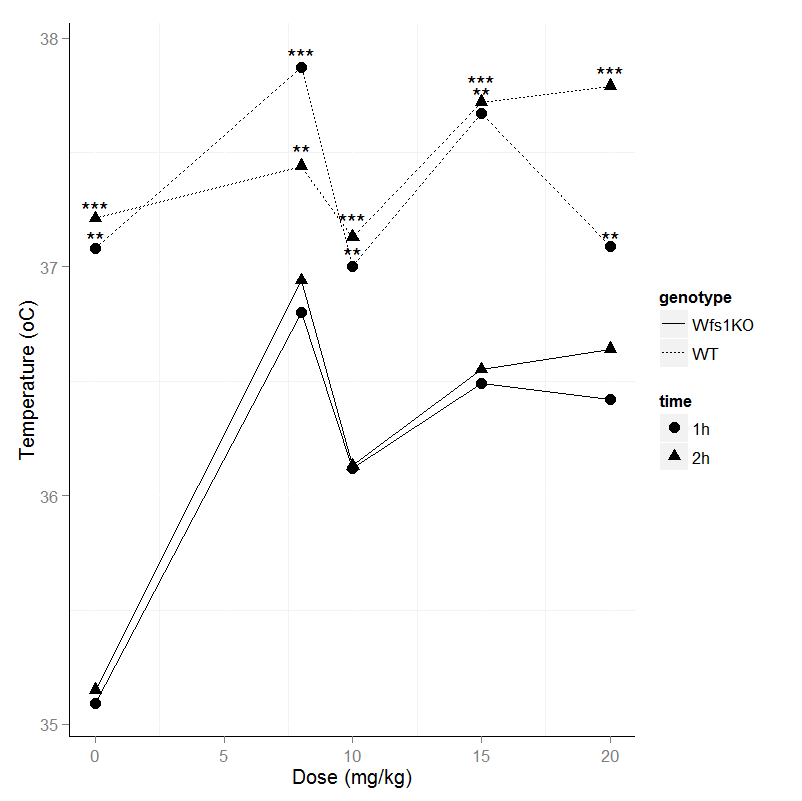

Supplement: Supplementary file 2 — Figure S1. Effect of menthol treatment on body temperature in Wfs1KO and WT mice. Different menthol doses did not change the body temperature of Wfs1KO mice and WT mice after 1 and 2 h of oral administration. Wfs1KO – body temperature (°C) of Wfs1‐deficient mice, WT – body temperature (°C) of wild‐type mice. Circle – mice rectal body temperature 1 h after menthol treatment, triangle – mice mice rectal body temperature 2 h after menthol treatment. *P < 0.05, **P < 0.005, ***P < 0.001. [file PHY2-4-e12660-s002.png]
